# Maternal Folic Acid Supplementation during Pregnancy and Childhood Allergic Disease Outcomes: A Question of Timing?

**DOI:** 10.3390/nu9020123

**Published:** 2017-02-09

**Authors:** Catrina L. McStay, Susan L. Prescott, Carol Bower, Debra J. Palmer

**Affiliations:** 1 Department of Health Western Australia, Perth 6004, Western Australia, Australia; Catrina.Mcstay@health.wa.gov.au; 2 School of Paediatrics and Child Health, The University of Western Australia, Subiaco 6008, Western Australia, Australia; Susan.Prescott@telethonkids.org.au; 3 Telethon Kids Institute, The University of Western Australia, Subiaco 6008, Western Australia, Australia; Carol.Bower@telethonkids.org.au; 4 Members of the in-FLAME International Inflammation Network, Perth 6000, Western Australia, Australia

**Keywords:** allergic disease, epigenetics, folate, folic acid, maternal diet, pregnancy

## Abstract

Since the early 1990s, maternal folic acid supplementation has been recommended prior to and during the first trimester of pregnancy, to reduce the risk of infant neural tube defects. In addition, many countries have also implemented the folic acid fortification of staple foods, in order to promote sufficient intakes amongst women of a childbearing age, based on concerns surrounding variable dietary and supplementation practices. As many women continue to take folic acid supplements beyond the recommended first trimester, there has been an overall increase in folate intakes, particularly in countries with mandatory fortification. This has raised questions on the consequences for the developing fetus, given that folic acid, a methyl donor, has the potential to epigenetically modify gene expression. In animal studies, folic acid has been shown to promote an allergic phenotype in the offspring, through changes in DNA methylation. Human population studies have also described associations between folate status in pregnancy and the risk of subsequent childhood allergic disease. In this review, we address the question of whether ongoing maternal folic acid supplementation after neural tube closure, could be contributing to the rise in early life allergic diseases.

## 1. Introduction

The dramatic increase in childhood allergic disease is now a serious public health issue in high-income countries [1,2,3,4]. Current evidence points to a multifactorial aetiology with a complex genetic predisposition, interacting with key environmental factors, including dietary changes. Both epidemiologic and mechanistic studies have linked specific and general consequences of recent dietary intake patterns, to biological effects in early life, including early immune dysregulation, an enhanced predisposition to inflammation, and inappropriate responses to normally harmless ubiquitous antigens, such as allergens, in very early life. The impact of diet is complex and is influenced through changing food sources and changes in specific nutrient intakes, with secondary immune and metabolic effects. These nutritional factors (including folate) have the potential to induce persistent developmental changes in gene expression through epigenetic modifications, and thereby, alter developing immune pathways, organ systems, and subsequent disease predisposition [5,6,7].

The developing fetus is highly responsive to environmental cues. One of the best described examples is the ability to modulate metabolic activity in response to the maternal nutrient supply—activating gene modules that conserve energy when maternal nutrition is restricted [8]. While these short term adaptations may provide short term advantages, they may also increase the risk of long term disease [8]. Some epigenetic modifications may also prompt early onset disease, such as allergic disease [9], although the evolutionary drivers of these adaptations are not well understood. In this context, there has been a focused interest on specific nutrients that are known to have direct effects on the epigenetic pathways that control gene expression during early development, such as folate.

Epigenetic mechanisms are finely coordinated processes that regulate the expression of genes, without alterations in the underlying gene sequence [10]. This includes DNA methylation, histone modifications, and non-coding RNAs, which coordinate gene expression to allow a responsive interface between the genes and the environment. Variations in the nutrient status can alter the epigenome, for example folate, which in addition to a myriad of biological actions, plays an essential role in the methylation of the universal methyl donor S-adenosylmethionine (SAM), and critically influences methionine metabolism pathways, protein synthesis and metabolism, cell multiplication, and tissue growth [11]. Changes in methyl donor activity of DNA methyl transferase enzymes, and an increased methylation of CpG sites of specific regions of DNA and associated changes in the chromatin structure, can silence gene transcription, thereby altering cellular activities [12].

Maternal nutrition is a critical factor, not only in providing the nutrient building blocks for fetal development, but also in steering the patterns of fetal gene expression that dictate the phenotype, patterns of metabolic and physiological responses, and future disease risk [13], even though subtle shifts in the profile of the nutrient supply. In this way, the epigenetic effects of maternal nutrition on fetal immune development are likely to be an important factor in the risk of allergic disease, particularly as differences in immune function can already be detected at birth, in those who go on to develop the disease [14]. This demonstrates lasting effects of in utero exposures on immune development, but also provides a window of opportunity for preventing immune-related diseases, and reversing the rising disease burden [7,15,16].

The hormonal milieu of pregnancy promotes tolerance at the materno-fetal interface, including immune regulatory responses and Type 2 T helper cell (TH2) responses, to protect the fetus from Type 1 T helper cell (TH1) alloantigen responses [17]. Fetal responses to allergens and other environmental exposures develop during this period, and are similarly TH2-skewed [17]. Specific regulatory responses in utero [18] have also been shown to be modified by the maternal environment [19]. Thus, variations in maternal environmental conditions may strongly set the scene for how the newborn responds to the postnatal environment, including the capacity to dampen inflammatory responses and to down-regulate TH2 responses in the postnatal period [20]. Disruption of these aspects of early immune regulation may interfere with normal TH1 maturation in the postnatal period, and lead to an allergic phenotype [21]. 

Of immediate relevance here are the antenatal effects of folate, shown to promote allergic predisposition in animal studies. Specifically, a landmark murine asthma model demonstrated that maternal folic acid supplementation promoted allergic airway disease in the offspring, by modifying DNA methylation and the expression of fetal genes associated with the development of an allergic phenotype [22]. Moreover, these epigenetic changes and the disease predisposition were inherited by the second generation. These biological actions of folic acid, together with epidemiological associations between infant allergic disease and maternal folate consumption in humans (below), raise questions on the unintended consequences of ongoing folic acid supplementation during pregnancy, potentially at higher than recommended requirements, after the window of risk for neural tube defects has passed.

Here, we examine world-wide changes in public health strategies, aimed at improving folic acid status over the past 25 years, and summarise the current evidence for a link between maternal folic acid supplementation during pregnancy and the development of childhood allergic diseases. This is based on a literature search for studies investigating the relationship between maternal folic acid supplement use in pregnancy, folate status, and childhood allergic disease outcomes; together with changes in folate- and folic acid-related public health strategies. The electronic databases of PubMed, Medline, Cochrane Library, and Google Scholar were searched, to identify articles published before the end of August 2016, using multiple combinations of the following search terms: allergic disease, atopy, epigenetics, DNA methylation, folate, folic acid, maternal diet, and pregnancy. Reference lists of recent review articles and key studies were also examined, to identify any other relevant studies not found during the initial database search. Multiple publications from some of the cohorts were examined, for potential overlapping participants and results. All relevant studies were included in this review.

## 2. Folate Requirements in Pregnancy

Folate requirements increase in pregnancy, in order to meet the maternal and fetal metabolic needs, and greater DNA synthesis and rapid cell division, during fetal development. An inadequate periconceptional maternal folate status has been associated with neural tube defects, such as spina bifida and anencephaly, highlighting the importance of entering pregnancy with adequate folate stores [23]. The international reference range for folate levels during pregnancy have been derived from 10 studies; seven studies relating to serum folate levels and three studies relating to red cell folate for the use of clinicians interpreting laboratory results [24]. These have been set using 5th to 95th percentile laboratory analyte values for red cell folate, for the first, second, and third trimesters of pregnancy, and are 137–589 ng/mL, 94–828 ng/mL, and 109–663 ng/mL, respectively; and 2.6–15.0 ng/mL, 0.8–24.0 ng/mL, and 1.4–20.7 ng/mL, respectively, for serum folate. The target for women of reproductive age is a level of red cell folate >906 nmol/L, to maximally reduce the risk of neural tube defects [23,25]. There is no target reference value for serum folate in women of reproductive age, due to a lack of epidemiological evidence for deriving these levels. Dietary reference values for folic acid intake during pregnancy vary internationally, with estimated daily average requirement values of 250 µg in the United Kingdom (UK); 370 µg in Japan; 400 µg in Europe; and 520 µg in Australia, United States (US), Canada and New Zealand.

## 3. History of Folic Acid Supplementation

Investigations into the therapeutic benefit of folic acid commenced in the 1930s, when Wills and colleagues investigated the treatment of tropical megaloblastic anaemia with yeast and marmite, and identified the presence of an unidentified bioactive factor [26]. The consumption of yeast extract, which contains folic acid, to nourish pregnant women, was also promoted from the 1930s [27]. In the 1950s and 1960s, researchers found that treatment with folic acid supplementation prevented megaloblastic anaemia in pregnancy [28,29]. Recommendations for utilising prophylactic folic acid treatment of pregnant women to prevent folate deficiency-related anaemia followed, and were implemented at a hospital located in Staffordshire (England) in around 1958, and in some ante-natal clinics in Britain by the late 1960s [29,30,31]. It was also recommended in the US, by the Manual of Standards in Obstetric-Gynaecologic Practice, from 1965.

Early research into folate deficiency in pregnancy and adverse pregnancy outcomes of fetal abnormalities, suggested a beneficial effect of folic acid supplementation in pregnancy and the risk of neural tube defect outcomes, only emerged in the 1960s [32,33]. Evidence of “periconceptional” folate status, emerged during the late 1980s and early 1990s [34,35,36,37,38,39]. A meta-analysis examining the protective effect of daily folic acid supplementation (alone or in combination with other vitamins and minerals) in preventing neural tube defects, when compared with no interventions/placebo or vitamins and minerals without folic acid in randomized trials, found a risk ratio of 0.28, and a 95% confidence interval of 0.15 to 0.52 [40].

During the past two decades, folate recommendations to prevent neural tube defects have been implemented in many regions of the world for women who are pregnant, planning a pregnancy, and of childbearing age. Early recommendations were made by the US in 1991, the UK in 1992, Canada in 1993, and Australia in 1994. Daily 400 µg folic acid supplementation recommendations for women of a reproductive age, and those planning a pregnancy, have now been adopted world-wide, and were endorsed by the World Health Organisation (WHO) in 2006.

## 4. Time Trends in Folic Acid Supplementation Practices

Folic acid supplementation rates have been found to vary over the last few decades. Public health education has been shown to increase the use of folic acid in planned pregnancy [41], although, research indicates that folic acid supplementation rates are increased amongst higher socioeconomic status (SES) groups, and that primary prevention strategies specifically targeting folic acid supplementation, even when supported with public health promotional activities, do not reach some socially disadvantaged groups, who are potentially at the greatest risk [42,43,44,45]. In particular, this includes younger women, those who are less educated, on low incomes, minority ethnic populations, and/or those with an unplanned pregnancy. A recent study from China supports this point. It found that pregnant women taking folic acid supplements after the first trimester, were more likely to be older, from urban areas, and have had a higher level of education, than those women who were not taking supplements in late pregnancy [46].

## 5. Dietary Intake of Folate from Food Sources

A recent systematic review and meta-analysis of dietary micronutrient intakes from food sources alone during pregnancy in developed countries [47], reported that the median dietary folate intakes (interquartile ranges) were 190 (190–232) µg/day for Australia/New Zealand, 334 (292–367) µg/day for the USA/Canada; 280 (260–315) µg/day for Europe, 217 (184–265) µg/day for the UK, and 276 (271–284) µg/day for Japan. Dietary intakes were consistent across the whole of the gestation for each geographical region. Importantly, estimated folate intakes were 13% to 63% less than each respective country’s dietary folate intake recommendations; reinforcing the need for pregnant women to take folic acid supplements.

## 6. Folic Acid Food Fortification

The limitations of relying on consumer-driven supplementation as the primary public health approach, led to the consideration of other folic acid-related policies, in order to achieve more equitable folate status in women of reproductive age. To address this, in addition to the existing folate recommendations and preventive health campaigns, some countries introduced voluntary and/or mandatory food fortification [48,49]; including developed economies such as Australia, Canada, Finland, Ireland, New Zealand, Norway, South Africa, the UK, and the US; and developing economies such as Brazil, Chile, China, and Costa Rica. Voluntary fortification of bread and breakfast cereal commenced in the UK from the mid to late 1980s. Both Canada and the US introduced similar mandatory fortification of all cereal flour food products into regulations in 1996, with the enactment of fortification by 1998. The US mandatory fortification requires all cereal grain to be fortified with folic acid, at 140 µg/100 g of grain food. Following these mandatory fortification initiatives, the effectiveness on the reductions in neural tube defect rates in the US and Canada, were around 50% and 54%, respectively [50]. Mandatory folic acid fortification of food has now been introduced in around 79 countries world-wide.

The governments of Australia and New Zealand implemented voluntary folate fortification of some foods in the mid-1990s, with manufacturers primarily fortifying breakfast cereals and bread products [49,51]. Following this, a 30% reduction in neural tube defects was reported in Western Australia; likely reflecting the combined influence of a folate promotion project and voluntary fortification measures [52]. Similar reductions in neural tube defects were reported in other countries, following mandatory folic acid fortification of staple foods, including Chile (43% reduction after 2000) [53], Brazil (35% reduction after 2004) [54], and South Africa (30% reduction) [55].

Evidence that the prevalence of neural tube defects was higher in Australia and New Zealand than the US, the UK, and Canada, led to the Australian and New Zealand Food Regulation Ministerial Council determination that neural tube defects posed a public health issue of sufficient severity, warranting a mandatory fortification approach. This led to the mandatory fortification of bread-making wheat flour with folic acid that came into effect in Australia in September 2009, with a two year phase-in period from 2007. A recent evaluation of this measure found significant reductions in the rates of neural tube defects of 14% in the states for which data were available, and 74% amongst the offspring of Australian Indigenous women in the same states. At this time, New Zealand delayed implementation of this food standard, mainly due to both industry and political issues [48]. Some countries, for example Sweden, have not introduced any fortification of foods, opting instead for a precautionary approach to minimise the risk of unintentional consequences [48].

## 7. Dietary Intake and Folate Status Post Folic Acid Food Fortification

In Australia, the aim of the mandatory folic acid fortification was to increase folic acid intake by 100 µg/day [56]. After the commencement of mandatory fortification, folic acid intake in the target population group of women of reproductive age, was estimated to have increased by 159 µg/day, when calculated using dietary modelling based on measured folic acid levels in bread samples in 2010 [56]. There was an expectation that folic acid supplementation would still be required to meet the required folate status for preventing neural tube defects [57]. The most recent Australian national dietary folate intake data, recorded by the Australian Health Survey in 2011/12, captured dietary intake of folate post fortification in Australia, including natural folate in food, and folic acid intake from fortified foods and supplements [58]. The mean daily intake for females aged 14–18 years, 19–30 years, and 31–50 years was 559.6 µg/day, 536.3 µg/day, and 529.4 µg/day, respectively. Dietary intakes were influenced by consumption of the fortified foods, which is dependent on the variations in dietary patterns of subgroups in the population. Current popular low carbohydrate diets, and low grain-based diets, may impact on some consumers’ fortified bread consumption [59], and hence lower their overall dietary folate intake.

The Australian Health Survey, conducted in 2011/12 [58], was the first national collection of biomedical results of folate status collected post implementation of mandatory folic acid fortification. Nutrient biomarker results reported median serum folate levels of 33.6 (interquartile range 26.5, 40.7) nmol/L, and mean red cell folate levels of 1601.0 (1366.0, 1848.4) nmol/L, for women of reproductive age (16–44 years of age). Nearly half of the female population surveyed (48.1%) had a serum folate level greater than 35.0 nmol/L; and 16.5% had a level greater than 45.0 nmol/L. These levels are in the upper range of the international reference range of 3–47 nmol/L [24]. Notably, a recent Canadian study found that folate levels post mandatory food fortification, were above the normal reference ranges for early and late pregnancy, and over twice the folate target range for women of reproductive age, with geometric mean (95% CI) red cell folate levels in early pregnancy (12–16 weeks) of 2417 nmol/L (2362, 2472), and late pregnancy (at delivery, 38–42 weeks) of 2793 nmol/L (2721, 2867) [60].

## 8. Timelines of Changing Folate Status and Increasing Prevalence of Allergic Diseases

**Figure 1:**
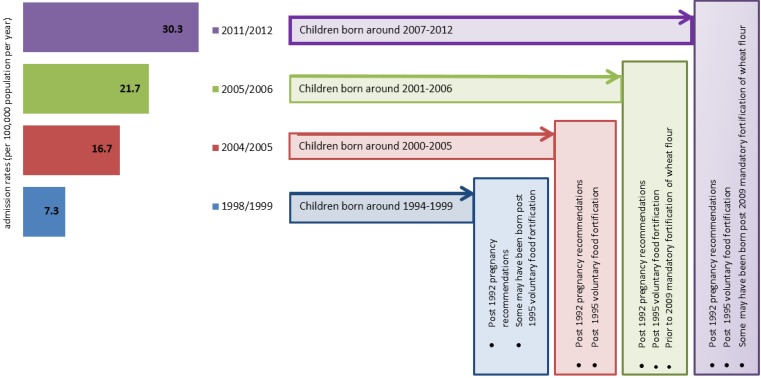
Food-related anaphylaxis hospital admissions rates (per 100,000 population per year) of children aged 0–4 years in Australia between 1998/99 and 2011/12, and the timing of Australian folic acid public health measures. Source: Adapted from Mullins, R.J. et al. [1].

The increased prevalence of childhood allergic diseases, including allergic rhinoconjuctivitis, eczema, and asthma, has been well-documented since the 1990s; although, for some countries that previously reported a high prevalence of asthma symptoms, the rates may now have plateaued or decreased [4,61]. This decrease in asthma prevalence is not consistent with the suggested role that folate supplementation may play in the development of asthma from animal studies. In a recent global survey of both developed and developing countries, the food allergy prevalence rates for children aged <5 years from nine countries with oral food, varied from 1% in Thailand, through to 10% in Australia, challenging proven food allergy data [4]. The rates of hospital emergency food-related anaphylaxis admissions have also increased in the UK, the US, and Australia, since 1990 [1]. The increase in food-related anaphylaxis admissions rates for 0–4 years old in Australia has occurred in parallel with the timeframes of implemented Australian folic acid related initiatives, although it should be highlighted that this does not imply any evidence of association (Figure 1).

## 9. Observational Studies Investigating the Relationship between Maternal Folic Acid Supplementation and/or Folate Status during Pregnancy and Childhood Allergic Disease

Ten publications [62,63,64,65,66,67,68,69,70,71] reporting the findings of the relationships between folic acid supplementation and/or folate status in pregnancy, and allergic disease outcomes in children, were identified. These studies encompassed folic acid supplementation use at differing time periods during pregnancy. To date, there have been no randomised, controlled trials investigating the effect of folic acid supplementation during pregnancy on childhood allergic disease outcomes. The observational studies differed in the investigated allergic disease outcomes; the methods used to determine the disease outcomes, such as clinical history (parental reported symptoms, parental reported diagnosis by medical practitioner, medical practitioner diagnosis); sensitization using skin prick tests and allergen specific IgE; and the “at risk” status of the children, due to the family history of allergic disease. The study time periods also cross decades; with differing levels of folic acid supplementation use, dietary folate intakes, and different folic acid food fortification policies. None of the studies were conducted in countries where there is a mandatory folic acid fortification in place.

### 9.1. Periconceptional and First Trimester Folic Acid Supplementation

The relationship between preconception and first trimester folic acid supplementation, or folate status in early pregnancy and allergic disease in children, has been investigated in two prospective mother and child birth cohort studies. In the Netherlands, the maternal plasma folate status at 13.5 ± 2.0 weeks’ gestation, of >16.2 nmol/L, was associated with and increased prevalence of atopic dermatitis outcomes in children up to 4 years of age [67]. This study recruited pregnant women residing in the Netherlands from the year 2000 onwards, at a time when dietary folate intakes may have been influenced by voluntary food fortification. A Norwegian study found that maternal-reported first trimester folic acid supplementation use, was associated with an increased risk of maternal-reported wheeze in children at 18 months of age [66]. Folate rich diets and folic acid supplementation in pregnancy recommendations, were the initiatives implemented in Norway during the study recruitment period.

### 9.2. Folate Status and/or Folic Acid Supplementation at Some Time during Pregnancy

In a recent Finnish study, folate intake and folic acid supplement use during pregnancy, were associated with an increased risk of a cow’s milk allergy in the offspring [70]. Finland had no voluntary folic acid fortification in place at the time of this study. In the Netherlands, folic acid use during pregnancy was associated with an increased risk of respiratory wheeze at the age of one year, but not at two-eight years; however, there was no association with other allergic diseases, such as asthma and eczema [62]. In a separate birth cohort study in the Netherlands, researchers found no association with asthma, wheeze, eczema, atopic dermatitis, and specific IgE sensitization, at multiple follow ups, between three months and six-seven years [69]; although, there was an association between higher maternal folate levels measured at around 35 weeks gestation, and a reduced asthma risk in offspring aged six-seven years. Dietary folate exposure may have been influenced by voluntary food fortification in The Netherlands at this time.

A South Korean cohort study of pregnant women at 12 to 28 weeks gestation, found that serum folate at a level of ≥9.5 ng/mL, was associated with a reduced risk of maternal-reported atopic dermatitis in offspring at 24 months; although, no relationship was found for late pregnancy (29–42 weeks gestation) [68]. This finding is contrary to the other studies described here, adding to the uncertainty of the potential role of folic acid supplementation on allergy development. This may reflect differences between countries. Also of interest in this study [68], is that higher serum folate levels were associated with increased levels of the immunoregulatory cytokine IL-10 in cord blood.

### 9.3. Second and Third Trimester Pregnancy Folic Acid Supplementation

Publications from those studies examining later pregnancy folate supplementation and allergic disease in the offspring outcomes, have also reported variable results (Table 1). These longitudinal studies provide evidence from cohorts of mother-child pairs across England, Norway, and Australia.

An English study found no association between maternal folic acid supplementation at 18 weeks and childhood atopy; although, there was some evidence of an association between maternal folic acid supplementation at 32 weeks and a child’s genotype (T allelle), and childhood atopy [64]. This is the earliest of the studies described here, where the cohort study time period occurred after voluntary permission to fortify bread and breakfast cereal in the mid/late 1980s, and prior to the 1992–1993 folic acid supplementation recommendations in the UK.

In a Norwegian cohort study, no association between late pregnancy folic acid supplementation use (after 12 weeks), and childhood allergic disease outcomes, was found; whereas, in a case control study nested in the same cohort, the risk of asthma was found to be increased in children at three years of age, with increasing quintiles of maternal plasma folate levels at 18 weeks [65,66]. Norway had dietary folate recommendations for pregnancy, but there was no fortification of food in place at the time of these studies.

Intriguingly, two Australian cohort studies found positive associations between folic acid supplementation in late pregnancy and the risk of asthma at 3.5 years [71]; and between higher doses of folic acid (>500 µg/day) from supplements in the third trimester and eczema at 12 months, along with a reduced risk of sensitization for fetal serum folate levels between 50 to 75 nmol/L after adjusting for confounders, including maternal allergy [63]. Only voluntary fortification of some food was implemented in Australia at the time of these studies.

The limitations of observational epidemiological studies should be highlighted here, given that the statistical significance of associations does not infer causality. Many of the observational studies were reliant on maternal-reported folic acid supplementation use and parental-reported allergic disease outcomes, which are subject to reporting bias. There is a lack of randomised clinical trials investigating the effect of the single dietary exposure of folic acid supplementation use during the second and third trimester of pregnancy. Randomisation of exposures is critical for accounting for the multiple confounding modifiable environmental factors that have been identified as potentially influencing the in-utero environment and related allergic disease outcomes in children, including other nutritional factors, such as vitamin D, gut microbiota, smoking, rural environments, pet keeping, and high SES-related health seeking behaviours [5,7,13,16].

## 10. Epigenetic Effect of Folate on Immune Gene Regulation

Following the hallmark study by Hollingsworth et al., in 2008, [22] demonstrating the transgenerational effects of folic acid on epigenetic regulation, and the predisposition to allergic disease, there has been intense interest in the effects of folate on immune development. In a randomised, controlled trial of healthy subjects (*n* = 20), where participants were allocated a daily folic acid supplement of 1.2 mg over a 12 week period (*n* = 10, 70% female), or a placebo (*n* = 10, 80% female), the folate status was found to influence the proteome, including the proteins involved in immune function [72]. Maternal use of folic acid supplements around the time of conception, has been found to be associated with increased methylation of genes in their children, such as insulin-like growth factor 2 (IGF2) in early childhood [73].

Recently, DNA methylation signatures from mononuclear blood cells, were found to predict clinical food allergy in infants aged 11–15 months [74]. Another study identified the longitudinal stable methylation differences in CD4+ T cells, from 12 month old infants with a diagnosed food allergy, highlighting the potential for epigenetic changes in these cells to influence the allergy phenotype via changes during T-cell development [75]. Interestingly, there is also evidence linking whole blood-isolated DNA methylation profiles and an active cow’s milk allergy; therefore indicating the potential of a link between the epigenetic changes in DNA methylation and the findings of the Finnish association study described above. In an epigenome-wide association study in US children, using DNA from whole blood samples, the methylation of gene loci was identified as being associated with a cow’s milk allergy [76]. A study of children in the Netherlands also found general hypermethylation in DNA regions, identified as being involved in immunological pathways of children, which challenge the proven cow’s milk allergy evidence, when compared to controls [77]. Mononuclear cells from children with a cow’s milk allergy have also been found to have distinct differences in the DNA methylation profiles for TH2- (IL-4, IL-5) and TH1(IL-10, IFN-γ)-associated cytokine genes, when compared to controls (healthy children) and tolerant children who had outgrown their previous cow’s milk allergy [78]. Collectively, these observations emphasise the need to better understand the impact of folic acid supplementation practices on aspects of fetal development, including the immune system. It is important to note the limitations of this review described above; and frame the current extent of the evidence base as being mainly derived from animal and observational epidemiological studies, with some emerging evidence from DNA methylation studies.

## 11. Conclusions

Folic acid acts as a methyl donor, with the capacity to alter the methylation of DNA. In animal studies, folic acid has been found to modify gene expression, linked to the development of allergic disease in offspring. These actions of folic acid, coupled with results from several human population studies, provide some foundation to bring into question the role of folic acid exposure in late pregnancy in the development of allergic disease in children, after the critical period of time for protection against neural tube defects. However, the epidemiological cohort study results are conflicting, and there is a significant lack of conclusive human trials, including in folate-replete populations. The likelihood of a multifactorial aetiology of allergic diseases in children, further highlights the need to examine this potential hypothesis using a randomised control trial study design, in order to better understand the clinical plausibility of this epigenetic role of folic acid supplementation in late pregnancy. Given the global trend of increasing allergic disease prevalence, this field of research is of interest to researchers, health practitioners, and food regulators alike; and has important research translation potential for informing food policy and health communication decision making.

## Figures and Tables

**Table 1:** Studies investigating the relationship between later pregnancy maternal folic acid supplementation and childhood allergic disease.

Reference	Study Design	Study Characterisitics	Key Allergic Disease Outcomes	Main Findings
Granell et al. 2008 [64] United Kingdom	Prospective cohort, pregnant women due date 1991–1992 with live births (*n* = 14,062); children aged 7–8 years (*n* = 5364).	Reported early and late pregnancy folic acid supplementation use; dietary folate intake at 32 weeks gestation, maternal and offspring methylenetetrahydrofolate reductase *(MTHFR)* C677T allele.	Skin prick test where positive SPT ≥ 2 mm weal; maternal reported child wheeze, and/or asthma diagnosed by physician.	No association between *MTHFR* C677T variant and atopy in children and maternal folic acid supplementation at 18 weeks and 32 weeks, or maternal folate intake at 32 weeks gestation.
Håberg et al. 2009 [66] Norway	Prospective cohort, pregnant women with children aged 18 months (*n* = 32,077) born 2000–2005.	Pregnant women reported maternal folic acid supplementation use in 1st trimester and after 1st trimester.	Maternal reported episodes of wheeze in children aged 18 months.	No association with folic acid supplement use after 12 weeks gestation and allergic disease outcomes.
Whitrow et al. 2009 [71] Australia	Prospective cohort, pregnant women (*n* = 557); mothers and their children aged 3.5 years (*n* = 490), 5.5 years (*n* = 423) recruited 1998–2000.	Pregnant women recruited in first 16 weeks of gestation. Reported food folate intake, inventory of supplement use at <16 weeks (early gestation) and at 30–34 weeks (late gestation).	Parental report of physician-diagnosed asthma of their child aged 3.5 and 5.5 years.	Late pregnancy folic acid supplementation use (weeks 30–34) was positively associated with risk of physician-diagnosed asthma at 3.5 years.
Håberg et al. 2011 [65] Norway	Case control, children aged 3 years (*n* = 1455 controls; 507 cases) born 2002–2004	Pregnant women reported maternal folic acid supplementation use after week 13 gestation; plasma folate at median gestation 18 weeks.	Maternal reported asthma diagnosis in children.	Asthma risk at 3 years increased in offspring with increasing quintile of maternal folate intake. Supplementation use associated with 2nd trimester folate status (Spearman correlation = 0.46).
Dunstan et al. 2012 [63] Australia	Prospective cohort, pregnant women (*n* = 628) with a history of allergic disease (*n* = 592); skin prick test positive (*n* = 615), children aged 12 months (*n* = 484), born 2002–2009	Maternal: food folate intake, folic acid supplement use in 3rd trimester. Calcuated dietary folate equivalants, serum folate; Cord blood: serum folate.	Medically diagnosed allergic diseases in infants aged 12 months of eczema and IgE mediated food allergy (symptoms on contact and sensitization by skin prick test).	Doses of folic acid > 500 µg/day from supplements in 3rd trimester associated with diagnosed eczema at 12 months. Reduced risk of sensitization for fetal serum levels between 50–75 nmol/L
